# Impact of the COVID-19 pandemic on maternal mental heath

**DOI:** 10.1192/j.eurpsy.2021.733

**Published:** 2021-08-13

**Authors:** E. Rose, M. Manoharan, J. Powell

**Affiliations:** 1 Psychiatric Intensive Care Unit, THE MAUDSLEY HOSPITAL, LONDON, United Kingdom; 2 Perinatal Mental Health, SOUTH LONDON AND MAUDSLEY NHS FOUNDATION TRUST, LONDON, United Kingdom; 3 Maudsley Simulation Team, SOUTH LONDON AND MAUDSLEY NHS FOUNDATION TRUST, LONDON, United Kingdom

**Keywords:** Perinatal Psychiatry, Covid-19, Maternal Mental Health, Coronavirus

## Abstract

**Introduction:**

As countries adopt strict quarantines and lockdowns, increasing attention has been given to the impact on mental wellbeing. The influence of this on perinatal mental health and service provision is important to consider, as these women may be particularly vulnerable to the negative effects already seen in general and psychiatric populations.

**Objectives:**

The impact on global mental health of Covid-19, and the isolation measures used to combat it’s spread, is increasingly acknowledged. We were interested in the effect the pandemic has had specifically on the mental health of women in the peripartum period. By reflecting on our experiences, we hope to generate ideas to improve services.

**Methods:**

We considered the effects of the pandemic in this high-risk population during each stage of contact with services. This included pre-conception, antenatal and postnatal periods, as well as the potential longitudinal and service effects. Recent case examples were identified and described from our busy and diverse South London perinatal psychiatry service.

**Results:**

Recent referrals to our service suggest the current crisis has been a key trigger for the deterioration of many women’s mental health. This includes women who have been impacted by various factors related to the pandemic, at all stages of the perinatal period.

**Conclusions:**

It is vital to maintain equality of access to perinatal services and to continue to consider how to deliver best care. This will involve adapting to the new working environment, and optimising care delivery using remote technologies where appropriate, in a way that is safe, accessible and acceptable to service users.

